# Editorial: Molecular chaperones and human disease

**DOI:** 10.3389/fmolb.2022.1068238

**Published:** 2022-11-17

**Authors:** Graham Chakafana, Stanley Makumire, Xolani Henry Makhoba

**Affiliations:** ^1^ Department of Chemistry and Biochemistry, Hampton University, Hampton, VA, United States; ^2^ Structural Biology Research Unit, Department of Integrative Biomedical Sciences, University of Cape Town, Cape Town, South Africa; ^3^ Department of Biochemistry, Microbiology, and Biotechnology, School of Molecular and Life Sciences, University of Limpopo, Sovange, South Africa

**Keywords:** molecular chaperone, HSP (heat shock protein), disease, proteostais, protein

Protein dysregulation is a hallmark of several human diseases. As such, molecular chaperones (being custodians of cellular proteostasis) are largely implicated in several diseases, including cancers and neurodegenerative disorders. The goal of this topic was to provide an update on the recent progress made in understanding the roles of molecular chaperones in the progression of model diseases with the prospects of targeting molecular chaperones toward the identification of novel drug therapies.

Heat shock proteins (Hsps) are some of the most studied molecular chaperones that are associated with several chaperonopathies. They particularly play an important role in the pathogenesis of malaria, which is caused by the parasite *Plasmodium falciparum*. *P. falciparum* exhibits a very complex life cycle where its development partly occurs in a poikilothermic mosquito and a homeothermic human host. Hsps, therefore, play an integral role in ensuring that proteostasis is maintained throughout the parasite’s life cycle and may thus be exciting antimalarial drug targets. Despite several efforts in the development of novel antimalarial therapies, drug resistance to front-line malarial treatments presents an urgent need for the development of novel therapies that are more reliable as highlighted by (Mrozek et al.). Using *in vitro* and cell-based assays, Muthelo et al. reported that the anti-cancer drug 2-Phenylthynesulfonamide (PES) exhibits antiplasmodial activity and capability to inhibit the functions of the *P. falciparum* cytosol-localized chaperones PfHsp70-1 and PfHop (Muthelo et al.). Barth et al. reviewed the current state of knowledge about the Hsp70 family of chaperones focusing on the suitability of these proteins and interactions for drug development (Barth et al.). Together with the Hsp90s, Hsp70 proteins have been widely studied in several disease models though, to date, there have not been many FDA-approved Hsp70 drugs. A review by Daniyan et al. looked into the roles of the exported parasite chaperone PfHsp70-x in the pathophysiology of cerebral malaria. The article also explored the possible links between host-parasite chaperones, and neurotransmitters, in relation to other molecular signalling components in the development of cerebral malaria (Daniyan et al.). This signalling pathway may provide further insights in antimalarial drug discovery. Collectively, the findings from these studies may contribute to the ongoing efforts in identifying novel antimalarial therapies, especially in the wake of growing parasite resistance against currently used drugs.

Chaperone/co-chaperone inhibition is being explored as a potential therapeutic target for several diseases. A review by Caillet et al. looks into the potential roles of molecular chaperones in mediating cell and systemic stress in COVID-19. The article discusses the roles of the host stress response as a convergent point for COVID-19 and several non-communicable diseases while further assessing the merits of targeting the host stress response to manage the clinical outcomes of COVID-19. It sets an interesting argument on the possible roles Hsps might play in COVID-19 and the potential of targeting Hsps in novel COVID-19 therapy. Using an *in silico* approach, Jamabo et al. conducted a structural analysis of the *Trypanosoma brucei* (*T. brucei*) Hsp90 variants in relation to human and other trypanosomal species. *T. brucei* is responsible for African trypanosomiasis which is a neglected tropical disease mostly endemic to sub-Saharan Africa. The parasite is spread by insects (tsetse fly). Similar to *P. falciparum*, the trypanosome relies on heat shock proteins for survival in the insect vector and mammalian host. In their analysis, Jamabo et al. identified a total of eighteen putative *T. brucei* Hsp90 co-chaperones with one notable absence being cell division cycle 37 (Cdc37) (Jamabo et al.). Their findings provide an updated framework for approaching Hsp90 and its interactions as drug targets in the African trypanosome (Jamabo et al.).

Hsp90 has previously attracted a lot of attention in drug discovery research and several Hsp90-targeting drugs have gone through various stages of clinical trials in the development of treatments for cancers and cardiovascular diseases. A study by Scalia et al. reported the reduced Hsp90 expression levels observed in a mutant version of the CCT5 subunit from a patient with distal motor neuropathy. This indicates that the imbalance of the chaperone has a negative impact which potentially triggers the development of distal motor neuropathy. Follow-up studies could provide further information on how Hsp-dysregulation triggers neurophysiological disorders. The role of Hsp90 in tumor progression and prognosis was also investigated in the development of small cell lung cancer (Huang et al.). Upon analyzing the relationship between eHSP90α expression and clinicopathological features, eHSP90α and NSE were found to be positively correlated in patients with small cell lung cancer (Huang et al.). This study provided new evidence for the efficacy response and prognostic assessment of SCLC with eHSP90α being suggested to be a potential SCLC biomarker.

Altogether, the articles included in this topic highlight the new advances that have been made in the application of molecular chaperones in translational medicine. The section also reported on the successes and potential use of Hsps in novel drug therapies and biomarkers for several disease models. As there has been rapid development in the field of chaperone biology, we envision that novel, groundbreaking findings will further contribute to the development of applicable solutions in drug and biomarker discovery.

